# Design and validation of an energy level diary for fatigue management in patients with post-COVID syndrome

**DOI:** 10.3389/fresc.2025.1633466

**Published:** 2025-07-21

**Authors:** Maryam Balke, René Garbsch, Jessica Cormann, Pantea Pape, Frank C. Mooren, Boris Schmitz

**Affiliations:** ^1^ Department of Early Neurological Rehabilitation, Cellitinnen Hospital St. Mary Cologne, Cologne, Germany; ^2^ Department of Rehabilitation Sciences, Faculty of Health, University of Witten/Herdecke, Witten, Germany; ^3^ DRV Clinic Koenigsfeld, Center for Medical Rehabilitation, Ennepetal, Germany

**Keywords:** post-COVID-19, post-COVID syndrome, chronic fatigue syndrome, diary approach, neurorehabiliation

## Abstract

**Background:**

Post-COVID syndrome (PCS) is a frequent condition with an incidence of 7.8–10.6 per 100 unvaccinated and 3.5–5.3 events per 100 vaccinated persons. Cognitive and motor fatigue are common clinical manifestations, limiting patients’ occupational, educational, and social activities severely.

**Objective:**

This study aimed to develop a diary to keep record of daily changes in energy levels of patients with PCS to adapt their rehabilitation program.

**Material and methods:**

We conducted a prospective observational study at two German rehabilitation centers in a codesign approach with repeated feedback loops. Daily energy changes were analyzed and validated using the Multidimensional Fatigue Inventory-20 (MFI-20).

**Results:**

The final diary revealed that morning and evening energy levels of patients with PCS differed significantly, with 49.6 ± 18.6% and 33.4 ± 19.7%, respectively (*p* ≤ 0.0001, on admission). Energy levels decreased by ∼5% with active therapy and increased by ∼5% with passive therapy (*p* < 0.0001). A comparison with MFI-20 at discharge showed good negative correlation (*r* = −0.5358, *p* < 0.0001), and patient interviews revealed that most patients (*N* = 19; 95%) rated the diary as “useful for self-reflection” and “helpful tool to learn the process of pacing.”

**Discussion/conclusion:**

This diary is a valid and user-friendly tool to detect and control the effects of daily therapy during the rehabilitation of patients with PCS. It will facilitate individual planning and adaptation of therapies in PCS and other fatigue groups and may help to implement an effective relation of exercise load to load capacity (pacing) for optimal coping with the disease and an improved handling of daily activities in patients’ lives.

**Clinical Trial Registration:**

Clinicaltrials.gov, identifier (NCT06883500).

## Introduction

Since the onset of the COVID-19 pandemic, rehabilitation facilities have been confronted with a large number of patients affected by post-COVID syndrome (PCS). Recently published data based on electronic health records revealed that PCS (or postacute sequelae of SARS-CoV-2 infection/PASC) among unvaccinated persons varies between 7.76 events per 100 persons (omicron era) and 10.64 events per 100 persons (pre-delta era). The cumulative incidence of PCS is lower in vaccinated persons, varying between 3.50 events per 100 persons (omicron era) and 5.34 events (delta era) (1). Per definition, PCS encompasses symptoms that persist for a duration exceeding 12 weeks after the onset of infection with the SARS-CoV-2 virus, beginning either during the infection or in the postinfection phase (2). Chronic cognitive and motor fatigue are common clinical manifestations of PCS, often accompanied by various other deficits such as muscle pain, headache, balance problems, autonomic dysfunction, sleep disturbance, and difficulties in alertness and memory (3–5). While a generally accepted definition of chronic fatigue syndrome does not exist, it can be defined as a subjective state that is beyond tiredness or sleepiness, interferes with activities of daily living and responsibilities, and is intensified by stress of any kind (6). Patients often describe their symptoms of fatigue as a lack of mental energy and muscle strength, with a delayed recovery after physical exertion (7). The situation is even aggravated if post-exertional malaise (PEM) is present, which is characterized by symptoms worsening after inadequate physical or mental activity. In this regard, PCS symptoms may overlap with clinical symptoms of myalgic encephalomyelitis/chronic fatigue syndrome (ME/CFS) (8).

Since patients’ occupational, educational, and social activities can be deteriorated to a comparable extent due to PCS and chronic fatigue as in other chronic diseases, such as multiple sclerosis or cancer (9), leading to a heavy economic burden (10), the need for effective medical rehabilitation is high (11).

As a method of energy self-management, the cognitive behavioral approach of “pacing” has been suggested to achieve symptom reduction and stabilization in ME/CFS (12, 13). Pacing aims to find a level of daily activity at which patients can function, but which does not increase symptom severity (14). The PACE trial highlighted that cognitive behavioral therapy and graded exercise therapy are strategies for efficient pacing (15), yet these results have been controversially discussed (16). A recent meta-analysis on activity pacing interventions for people with chronic fatigue syndrome showed promising results in that interventions were effective at reducing fatigue, psychosocial distress, physical function, and depression (17). However, other authors call for additional RCTs using objectively quantified and digitized pacing over longer durations since the methods of pacing are not standardized (18). In this regard, a retrospective analysis of 86 patients with PCS demonstrated that a stronger adherence to pacing, as measured by the engagement in pacing subscale (EPS), leads to faster recovery and improvement of symptoms (19). Our observations align with current recommendations that both overexertion and underexertion should be avoided in the case of PCS. Therefore, graded exercise therapy, for example, cannot be applied in this context (20).

A diary is a useful tool to reflect and document an individual's health experience daily over a given time (21), so that symptom and behavioral trends become visible with less recall bias than in questionnaires or retrospective interviews alone (22). In the medical field, diaries are a means of clinical evaluation of behavior and efficacy of therapies, e.g., in sleep medicine (23), oncology (24, 25), urinary incontinence (26), Parkinson's disease (27), headache (28), seizure (29), and psychological implications of intensive care (30, 31). Moreover, since the onset of digital applications, it has become easier to track human behavior, to draw conclusions on the validity of existing theories, and to provide big data, as shown successfully with Cure (32) and MyFitnessPal app (33). Recently, a digital application targeting patients with chronic fatigue syndrome has been launched (34). A validated PCS-specific diary—developed in collaboration with patients with PCS—is currently not available for both patients and therapists.

The present study aimed to develop a diary together with affected patients to keep record of patients with PCS' energy levels throughout a structured rehabilitation process. We hypothesized that the diary would be capable of documenting the effects of different therapies on daily energy levels and would help to adapt therapies more specifically to patients’ needs.

## Methods

### Study design and patients

A prospective multicenter observational cohort study of patients with PCS referred for medical rehabilitation was performed according to STROBE guidelines at Clinic Koenigsfeld Ennepetal, Germany, and Neurological Therapy Center Cologne, Germany, between January 2022 and April 2024. In an initial step, a diary was developed by experts together with patients (codesign; see below). Versions of the diary were provided to patients to investigate usability and sensitivity to daily changes in fatigue/energy levels. Patient interviews were performed to collect feedback. The final version of the diary was validated against the Multidimensional Fatigue Inventory-20 (MFI-20). Patients completed the diary during inpatient (Koenigsfeld) and outpatient (Cologne) medical rehabilitation, both including a combination of physical, cognitive, and respiratory training as well as relaxation and psychotherapy as indicated. Inclusion criteria were a history of at least one COVID-19 infection (positive PCR test at the time of infection) and ongoing or newly expressed performance deficits lasting for at least 3 months prior to recruitment. Performance deficits were documented according to the recent consensus statement, with the cluster of lead symptoms including fatigue/exercise intolerance, shortness of breath, and cognitive dysfunction (35). Patients with coronary artery disease (CAD) after acute myocardial infarction or reperfusion via coronary angioplasty or coronary artery bypass graft, who completed and used the fatigue diary as part of a prospective cohort study, were included as a control group.

### Energy diary—phases of development

A codesign approach was used, and the diary was developed over three phases through repeated feedback loops, including interviews with patients with PCS and an expert consensus, consisting of doctors, scientists, and therapists, using the Delphi method.

#### Phase 1A and 1B: expert consensus and diary development

An expert panel of clinicians, nurses, therapists, and scientists was built to create a specific diary for patients with PCS during rehabilitation, which should be able to detect energy fluctuations throughout the therapy day. In an initial step, existing fatigue diaries for patients’ self-assessment were screened for similarities and applicability in PCS (36–39). The requisition was that the format would be applicable for rehabilitation settings and would be comprehensive enough to reflect intraday changes of fatigue/energy levels correctly. Version 1 of the diary asked for the self-reporting of fatigue at the beginning and at the end of the rehabilitation day, using a visual analog scale from 0 (no fatigue) to 10 (maximum fatigue) (“please indicate your level of fatigue at the beginning and at the end of the day on a scale between 1 and 10”). Emojis ranging from happy to sad, colored from green to red, were used to assess patients' overall condition after each exercise. Participants received a folder containing a diary, user instructions, and legend, comprehensive information about pacing and space for personal notes, and an introduction to the concept by a therapist. The usability assessment of Version 1 involved 27 patients. During weekly visits, clinicians asked about patients' experiences and feedback, which was reported to the panel of experts.

Based on the experiences and feedback on Version 1, an adapted Version 2 of the diary asked for “energy levels” instead of “fatigue” at the beginning of the day, after each therapy session, and at the end of the day, using a visual analog scale from 0 (no energy) to 100 (maximum energy) (“please indicate your level of energy at the beginning of the day, after each therapy session, and at the end of the rehabilitation day on a scale between 0 and 100”; Supplementary Figure 1). The assessment of the usability of Version 2 involved 20 patients.

#### Phase 2: usability and qualitative content analysis interviews

Feedback on wording and overall usability of the diary Version 2 was collected via standardized interviews with patients. Twenty patients with PCS took part in individual semi-structured problem-centered phone-based interviews (guided by JC), including nine open-ended questions (Supplementary Table 1). Answers were anonymized, transcribed, coded, and categorized using qualitative content analysis in accordance with the established classification system by Mayring (40–42). Interpretation involves deciphering and contextualizing the coded text passages in relation to the research question, elucidating patterns, connections, and meanings. The results were presented to the expert group to decide on necessary modifications and the design of the final version.

#### Phase 3: validation and clinical applicability

The final version of the fatigue diary was validated in *N* = 66 patients using the Multidimensional Fatigue Inventory (MFI-20) (43), which provides an aggregate score and three subscales pertaining to general, physical, and mental fatigue (scores range from 0 to 100, with higher values indicating elevated levels of fatigue). The mean self-reported energy levels were correlated with MFI-20 scores quantified on admission and discharge. The effects of different therapy categories on changes in self-reported energy levels were assessed by grouping therapies performed during inpatient rehabilitation (Koenigsfeld, *n* = 33) into three categories: active therapies, consisting of physical (endurance training, strength training, outdoor activities, etc.) and respiratory therapies (respiratory muscle training), cognitive therapies (cognitive training, education and talks, etc.), and passive therapies (relaxation, massages, hydrojet, etc.).

### Statistical analysis

Data were analyzed using SPSS (V.28, IBM, Armonk, USA) and GraphPad Prism (V.10, GraphPad Software, Boston, USA). Constant variables are expressed as mean ± standard deviation (SD) or 95% confidence interval (CI) or median (range) as indicated. The categorical variables are presented as *n* (%). Energy levels in the morning and evening were averaged across all patients for analysis of daily changes. Energy levels at admission and discharge were defined as the mean of the morning and evening energy levels closest to admission and discharge, within a tolerance of 3 days (in case of missing data). For comparisons of energy levels, only values in which at least 20% of the cohort were involved were used. Effects of different therapy types were analyzed only from center Koenigsfeld for better comparability. Non-normal distribution was tested using skewness and kurtosis. Differences between groups were analyzed using mixed-effects model, unpaired two-sided *t*-test, or Mann–Whitney *U* test if indicated. Chi-square test was used for categorical variables. Differences within-group were analyzed using mixed-effects model, paired two-sided *t*-test, or Wilcoxon test in case of non-normal distribution. Spearman rank correlation analyses were performed to investigate correlations between energy levels and MFI scores. Differences in subgroups [sex and age (divided by median split)] over time were analyzed using two-way repeated-measures ANOVA. Statistical significance was accepted at *p* < 0.05.

### Ethics approval and consent to participate

The study was approved by the respective ethical review committee (Ethik-Kommission Universität Witten/Herdecke; reference number 159/2021 for PCS and 115/2020 for CAD patients) and conformed to the Declaration of Helsinki. Written informed consent was obtained from all participants for study participation.

## Results

### Phase 1A and 1B: expert consensus and diary development

**Figure 1:**
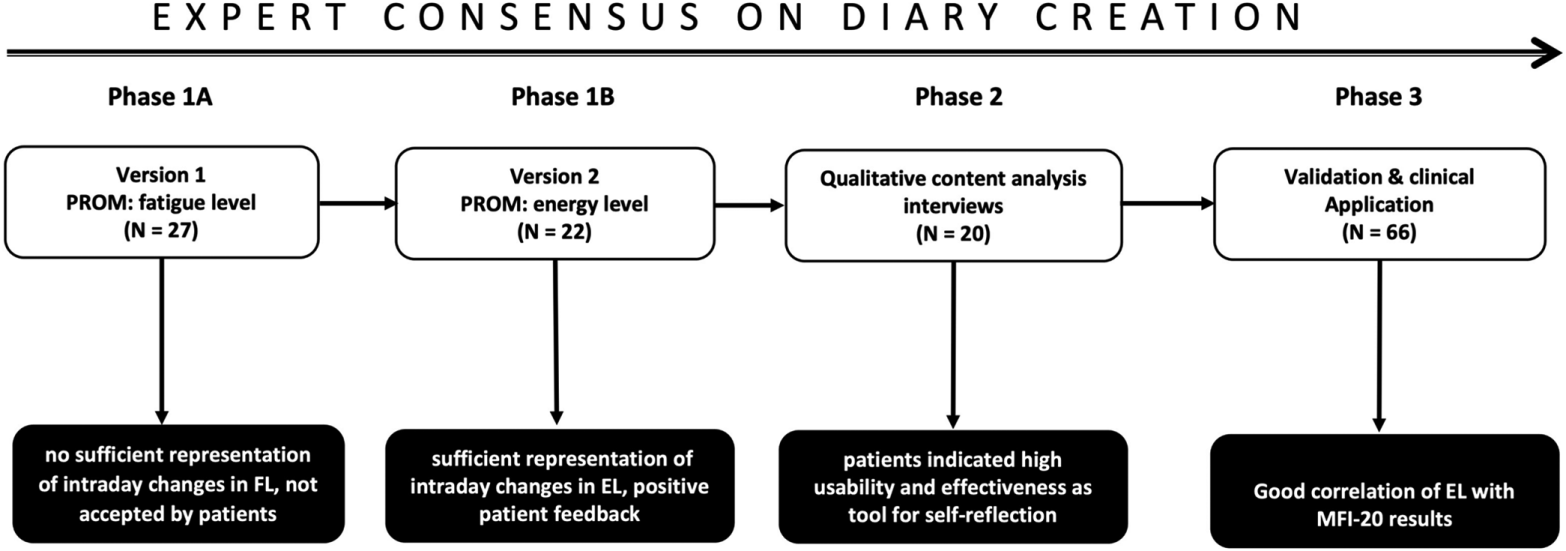
Expert consensus on diary creation, validation, and clinical application. The process of design, validation, and testing of clinical applicability was structured in three phases involving both an expert panel and affected patients. Phase 1 (A/B) Comprised monthly interdisciplinary expert meetings and initial patients’ feedback. Diary Version 1 asked for changes in FL, but daily variations were not reflected, and patients rejected Version 1. Final result: Version 2 reflects intraday changes in energy levels. Phase 2: Qualitative content analysis and usability assessment of Version 2 using structured patient interviews. Final result: sufficient representation of intraday changes in EL, positive patient feedback. Phase 3: Validation and testing of clinical application. Correlation of mean daily energy levels with results of the Multifunctional Fatigue Inventory-20. Investigation of the effects of therapy sessions on energy levels. Result: good correlation with energy level, useful to monitor and control effects of individual therapy sessions. PROM, patient-reported outcome measure; FL, fatigue level; EL, energy level. White box: activity in expert consensus/codesign. Black box: results of expert consensus process.

Figure 1 provides an overview of the diary creation process including final validation. Version 1 of the diary asked patients to indicate intraday changes in fatigue on a visual analog scale from 0 to 10 (0 = no fatigue, 10 = maximum fatigue). Evaluation of results from 27 patients (81.5% women, 18.5% men, mean age F:M = 42 ± 13.0:49 ± 13.0) revealed that the diary was not able to detect changes in patients' fatigue levels over the day (morning vs. evening levels), so that an evaluation of therapy effects would not be possible. Based on patients' feedback and discussion in the expert panel, Version 2 of the diary asked for self-reported changes in “energy levels” instead of “fatigue levels.” This version was sensitive to daily changes in energy levels and also reflected the effects of different therapy sessions (see below, Phase 3: validation and clinical applicability).

### Phase 2: qualitative content analysis interviews

Twenty patients (70% women, 30% men, mean age F:M 47.6 ± 14.1:45.6 ± 16.0) were interviewed on wording and general usability of the diary (Version 2) using nine open-ended questions (Supplementary Table 1). Overall, patients rated the diary as having appropriate wording and being practical to use. The majority of patients (*N* = 19, 95%) indicated that formatting and color features were adequate and considered the diary useful as a tool for self-reflection and as a guide and memory aid. Percentual tracking (energy level 0–100) was considered as helpful to assess one's energy levels by 11 patients (55%), while visual tracking with smileys was rated as helpful by 4 patients (20%), and 3 patients (15%) indicated percentual tracking and visual tracking as equally helpful for the subjective assessment of energy levels. In addition, most patients (*N* = 19; 95%) considered the diary a helpful tool to learn the process of pacing, understand the development of fatigue, and follow therapy effects. Fifty-five percent indicated that a digital diary version (delivered via an app) would be preferable. Analysis of the interviews in the expert consensus process led to the conclusion that no major changes were required. However, one extra page was added to each week for individual notes and comments.

### Phase 3: validation and clinical applicability

Version 2 of the fatigue diary with subjective assessment of energy levels was provided to 80 patients with PCS to be filled out during the rehabilitation process. Ultimately, diaries of 66 patients were available for analysis (*N* = 7 not handed in on discharge, *N* = 6 premature discharge, *N* = 1 not usable, incorrectly completed, Figure 1).

### Patients' characteristics

Patients with PCS (*N* = 66; 67% women) involved in the final usability and validation study were referred to rehabilitation with an average age of 51.7 ± 10.3 years and a mean time interval between first infection and start of medical rehabilitation of 453.0 ± 294.7 days (Table 1). Fatigue/exercise intolerance was observed in ∼96% of patients, while cognitive dysfunction (∼76%) and shortness of breath (∼59%) were less common. Detailed examination of fatigue using the MFI-20 questionnaire revealed overall high levels of fatigue (MFI-20 score, 78.0 ± 12.4), with as many as 52% of patients with PCS exceeding the threshold for chronic fatigue syndrome (>70; Table 2). During the acute phase of infection, 97% of patients received ambulant care or acute care at home, while 3% of patients required in-hospital care. Overall, patients reported a high frequency of circulatory system disorders as well as endocrine, nutritional, and metabolic disorders.

**Table 1:** Anthropometric and clinical data, medication, and blood parameters.

Variable	Overall (*n* = 66)	Ennepetal (*N* = 33)	Köln (*n* = 33)	*p*-value
Anthropometric data				
Age, years	51.7 ± 10.3	55.9 ± 6.5	48.6 ± 12.4	**0.012**
Sex, *n* (%)				**0.018**
* Female*	44 (66.7)	17 (51.5)	27 (81.8)	—
* Male*	22 (33.3)	16 (48.5)	6 (18.2)	—
Height, cm	171.8 ± 10.1	172.8 ± 10.6	170.8 ± 9.6	0.421
Weight, kg	86.3 ± 20.5	93.8 ± 20.1	78.8 ± 18.3	**0.002**
BMI, kg*m^−2^	29.3 ± 7.2	31.6 ± 7.8	27.0 ± 5.7	**0.007**
(Post) COVID-19 characteristics				
Acute care during infection				0.492
* Ambulant care*	64 (97.0)	31 (93.9)	33 (100.0)	—
* Hospitalized, w/o ventilation*	2 (3.0)	2 (6.1)	0 (0)	—
* Hospitalized, with ventilation*	0 (0)	0 (0)	0 (0)	—
Time between acute infection and rehabilitation start	453.0 ± 294.7	412.1 ± 245.8	493.9 ± 335.5	0.263
Lead symptoms at rehabilitation start				
*Fatigue/exercise intolerance*	63 (95.5)	30 (90.9)	33 (100.0)	0.238
*Shortness of breath*	39 (59.1)	28 (84.8)	11 (33.3)	**0.001**
*Cognitive dysfunction*	50 (75.8)	26 (78.8)	24 (72.7)	0.775
Comorbidities				
Diseases of the circulatory system, *n* (%)	36 (54.5)	25 (75.8)	11 (33.3)	**0.001**
*Arterial hypertension*	27 (40.9)	17 (51.5)	10 (30.3)	0.132
*Other*	15 (22.7)	13 (39.4)	2 (6.1)	**0.002**
Endocrine, nutritional, or metabolic diseases, *n* (%)	32 (48.5)	22 (66.7)	10 (30.3)	**0.006**
*Obesity*	22 (33.3)	15 (45.5)	7 (21.2)	0.066
*Hypothyroidism*	13 (19.7)	4 (12.1)	9 (27.3)	0.215
*Hyperlipidemia*	7 (10.6)	6 (18.2)	1 (3.0)	0.105
*Type 2 diabetes mellitus*	6 (9.1)	5 (15.2)	1 (3.0)	0.197
*Other*	5 (7.6)	5 (15.2)	0 (0)	0.053
Diseases of the musculoskeletal system and connective tissue, *n* (%)	25 (37.9)	10 (30.3)	15 (45.5)	0.310
Mental and behavioral disorders, *n* (%)	20 (30.3)	9 (27.3)	11 (33.3)	0.789
*Depressive/adjustment disorders*	9 (18.2)	3 (9.1)	6 (18.2)	0.475
*Other*	12 (18.2)	6 (18.2)	6 (18.2)	1.000
Diseases of the nervous system, *n* (%)	17 (25.8)	10 (30.3)	7 (21.2)	0.574
*Migraine/headache*	6 (9.1)	1 (3.0)	5 (15.2)	0.197
*Other*	11 (16.7)	9 (27.3)	2 (6.1)	**0.044**
Diseases of the respiratory system, *n* (%)	15 (22.7)	8 (24.2)	7 (21.2)	1.000
Diseases of the digestive system, *n* (%)	4 (6.1)	2 (6.1)	2 (6.1)	1.000

Data are presented as mean ± SD or *n* (%). Between-group comparison was performed using unpaired two-sided *t*-test, Mann–Whitney *U* test, or chi-square test. Diseases/symptoms with a prevalence <5% are not reported. BMI, body mass index.

Bold values indicate significant differences.

**Table 2:** Fatigue assessed by Multidimensional Fatigue Inventory-20 (MFI-20).

Time point/ change	Overall (*n* = 66)	Ennepetal (*n* = 33)	Köln (*n* = 33)	*p*-value
Overall score				
T0	78.0 ± 12.4	75.9 ± 12.8	80.4 ± 11.8	0.153
T1	72.3 ± 16.8	69.0 ± 17.1	76.2 ± 15.8	0.095
*Δ*	−5.6 ± 12.9**	−6.9 ± 13.7	−4.1 ± 11.9	0.418
General fatigue				
T0	89.4 ± 12.9	87.1 ± 13.8	91.9 ± 11.5	0.147
T1	81.5 ± 17.4	78.3 ± 16.9	85.2 ± 17.7	0.129
*Δ*	−8.0 ± 14.7****	−8.8 ± 14.4	−7.0 ± 15.2	0.634
Physical fatigue				
T0	83.8 ± 16.9	81.4 ± 13.8	86.6 ± 19.7	0.242
T1	76.9 ± 21.3	72.4 ± 20.9	82.1 ± 20.8	0.075
*Δ*	−6.9 ± 15.4***	−8.9 ± 16.1	−4.5 ± 14.4	0.261
Mental fatigue				
T0	76.9 ± 17.3	76.4 ± 16.6	77.6 ± 18.2	0.785
T1	72.5 ± 18.7	69.4 ± 18.7	76.3 ± 18.2	0.154
*Δ*	−4.3 ± 18.3*	−7.0 ± 17.5	−1.3 ± 18.9	0.229

Fatigue was assessed by the Multidimensional Fatigue Inventory-20 (MFI-20 questionnaire) at admission (T0) and before discharge (T1). Data are presented as mean ± SD. Between-group and within-group comparisons were performed using mixed-effects model. MFI-20: range 0–100 (higher = greater fatigue).

*Δ* (delta) indicates the absolute difference between T0 and T1.

* *p* < 0.05.

** *p* < 0.01.

*** *p* < 0.001.

**** *p* < 0.0001 significantly different from T0 to T1 (all *p* ≤ 0.0275).

### Diary results—intraday energy level changes

**Figure 2:**
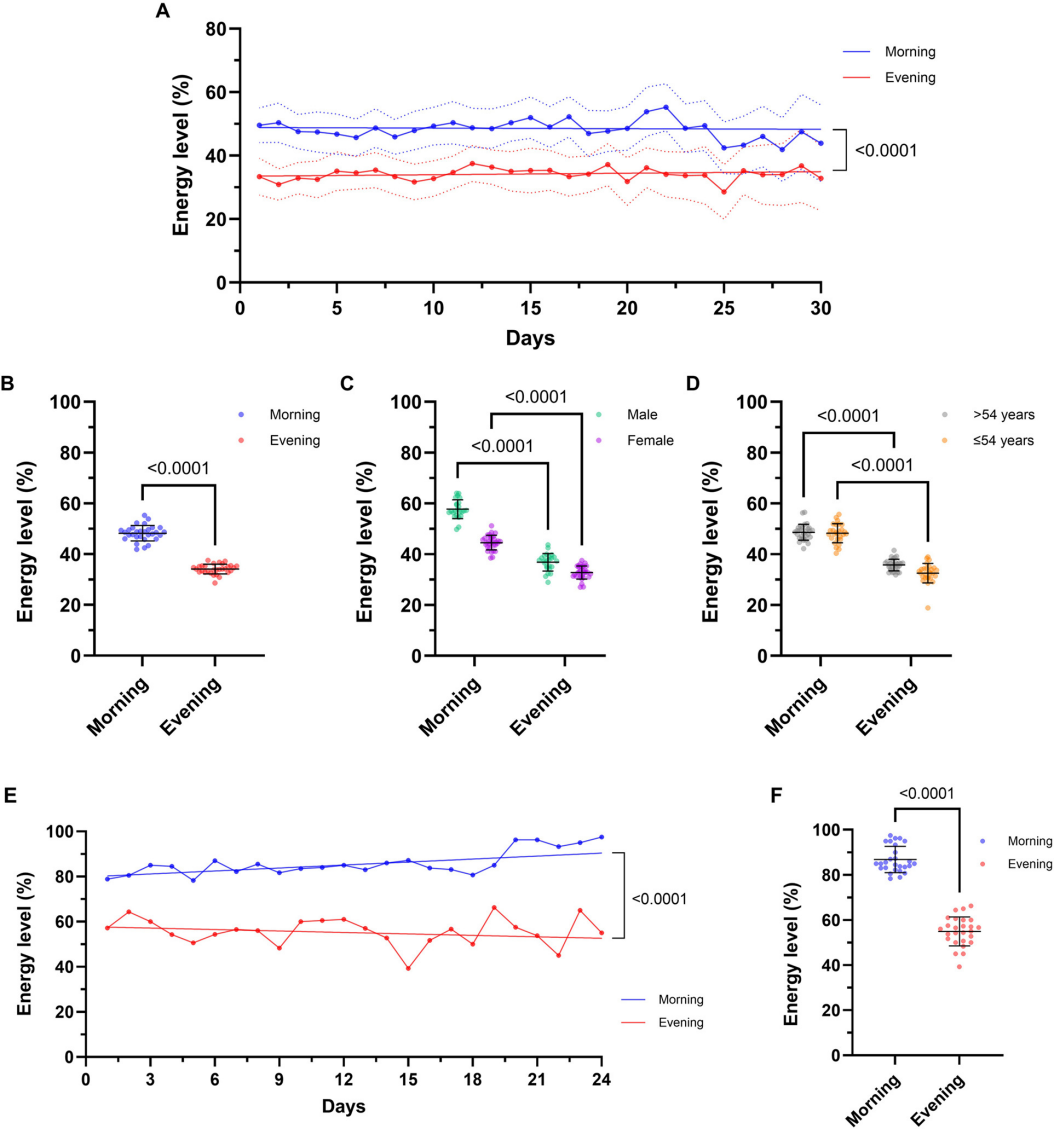
The post-COVID-19 syndrome (PCS) energy diary registers changes in patients’ daily energy levels during rehabilitation. Energy levels of PCS patients (*N* = 66) are given **(A)** over the entire rehabilitation process per day (morning vs. evening), **(B)** in comparison between all morning and evening ratings and separated by **(C)** sex (female, *N* = 44; male, *N* = 22) and **(D)** median age (>54 years, *N* = 31; ≤ 54 years, *N* = 35). Energy levels of patients with coronary artery disease (CAD; *N* = 11) are given **(E)** over the entire rehabilitation process per day (morning vs. evening) and **(F)** in comparison between all morning and evening ratings. Comparison between morning and evening ratings reveals that the fatigue diary documents the loss of energy during the day, as well as energy restoration overnight (*p* < 0.0001), for both PCS and CAD patients. No differences in morning and evening energy levels were seen from admission to discharge (both *p* > 0.05). Significant difference in morning and evening ratings was seen, independent of sex and age (all *p* < 0.0001). Data are presented as the mean of individual data points (with 95% confidence interval) or as mean ± SD. Two-way repeated-measures ANOVA was performed to analyze morning and evening ratings (averaged across all patients) over the entire rehabilitation process as well as for subgroup analyses. Comparison of morning and evening ratings was performed using paired *t*-test. The trend line was modeled using linear regression slopes.

Morning and evening energy levels differed with a significant decrease during the day and a significant increase with overnight recovery (both *p* ≤ 0.0001; Figures 2A,B). On admission, patients reported a mean energy level of 49.6 ± 18.6% in the morning (max. 100%; min. 15%) and 33.4 ± 19.7% in the evening (max. 80%; min. 20%) using the diary. Over the course of rehabilitation, patients showed small fluctuations in both morning (mean max. 55%; min. 42%) and evening (mean max., 38%; min., 29%) energy levels, but no statistically significant changes were seen from admission to discharge (both *p* > 0.05). A difference in morning and evening energy levels was seen independently of sex and age (both *p* < 0.0001) (Figures 2C,D). Of note, male and female patients with PCS indicated significantly different morning energy levels (male, 57.7%; female, 44.5%; *p* < 0.0001). In addition, on admission, 17.2% of patients with PCS were affected by an energy level of ≤10% in the evening, which improved by the time of discharge, leaving only 9.5% of patients in this state (*p* = 0.222). Compared to PCS patients, CAD patients (*N* = 11, 57.1 ± 5.3 years, 55% women) had significantly higher morning (86.8 ± 5.8 vs. 48.2 ± 3.1) and evening (54.9 ± 6.4 vs. 34.1 ± 1.9) energy levels (both *p* < 0.0001), while the significant daily energy reduction was also seen in CAD patients (Figures 2E,F). During rehabilitation, morning energy levels of CAD patients increased slightly (+7.5, n.s.), while energy levels of PCS patients remained stable. None of the CAD patients reported an energy level of <10% in the evening.

Analysis of inpatient vs. outpatient rehabilitation revealed significantly different mean morning energy levels (inpatient, 57.6 ± 4.4%; outpatient, 39.9 ± 3.6%; *p* < 0.0001), while evening energy levels were comparable (inpatient, 33.5 ± 4.2%; outpatient, 34.5 ± 3.3%; *p* > 0.05). This difference was detected in both, female (inpatient, 54.4 ± 6.6%; outpatient, 38.9 ± 3.2%; *p* < 0.0001) and male (inpatient, 61.3 ± 4.4%; outpatient, 45.9 ± 8.6%; *p* > 0.0001) patients, suggesting that inpatient rehabilitation may allow a larger energy gain after daily therapies have been completed (all *p* ≥ 0.05; Table 2).

### Diary validation and effects of therapy on energy levels

**Figure 3:**
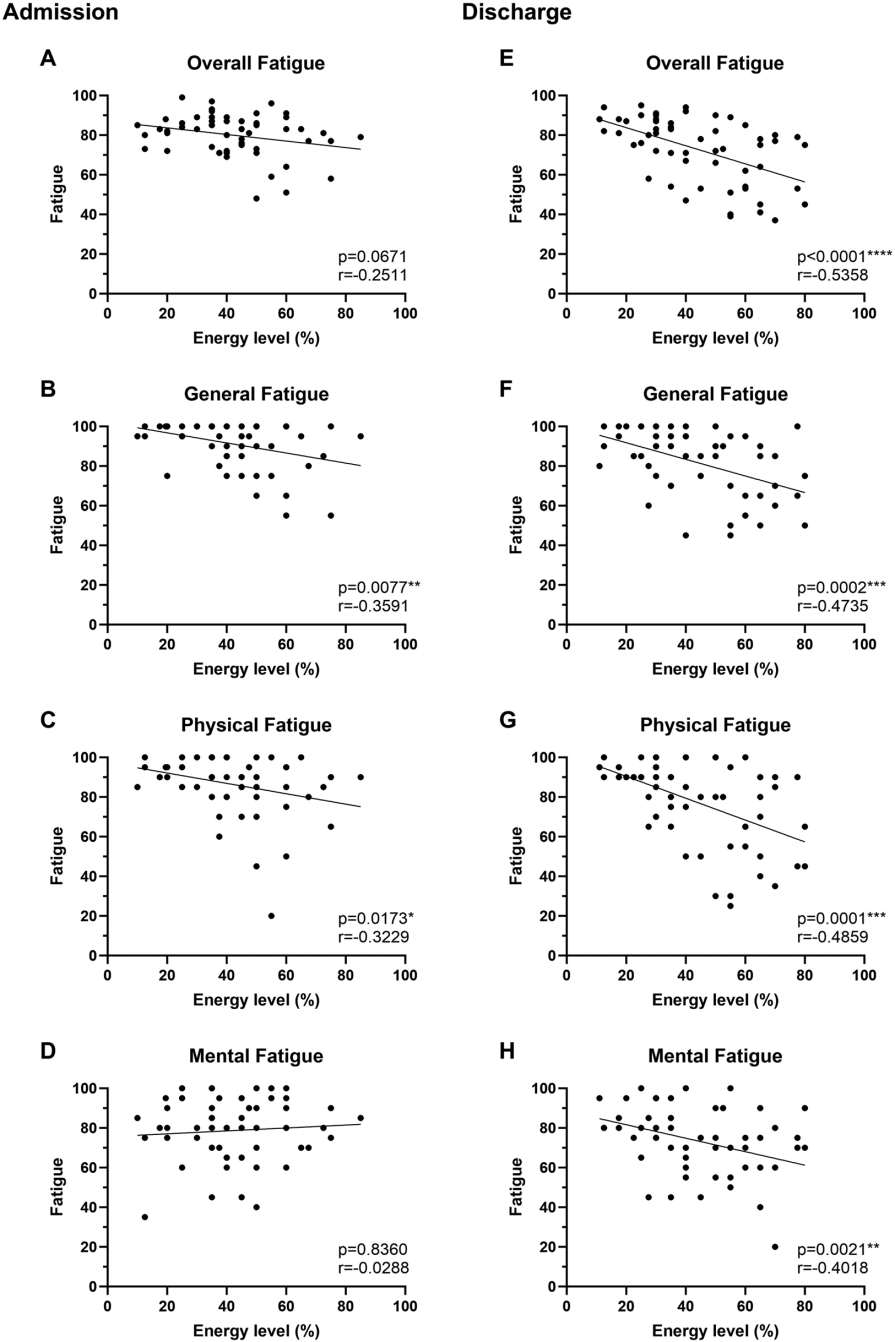
Diary energy levels correlate with Multidimensional Fatigue Inventory-20 (MFI-20) scores. Fatigue of PCS patients was assessed by MFI-20 (score 0–100, higher values = higher fatigue) at admission **(A–D)** and before discharge **(E–H)** and compared with mean daily energy levels assessed by the fatigue diary (*N* = 58). Each data point represents an individual measurement. Correlations were performed using Spearman rank correlation. The trend line indicates linear regression.

**Figure 4:**
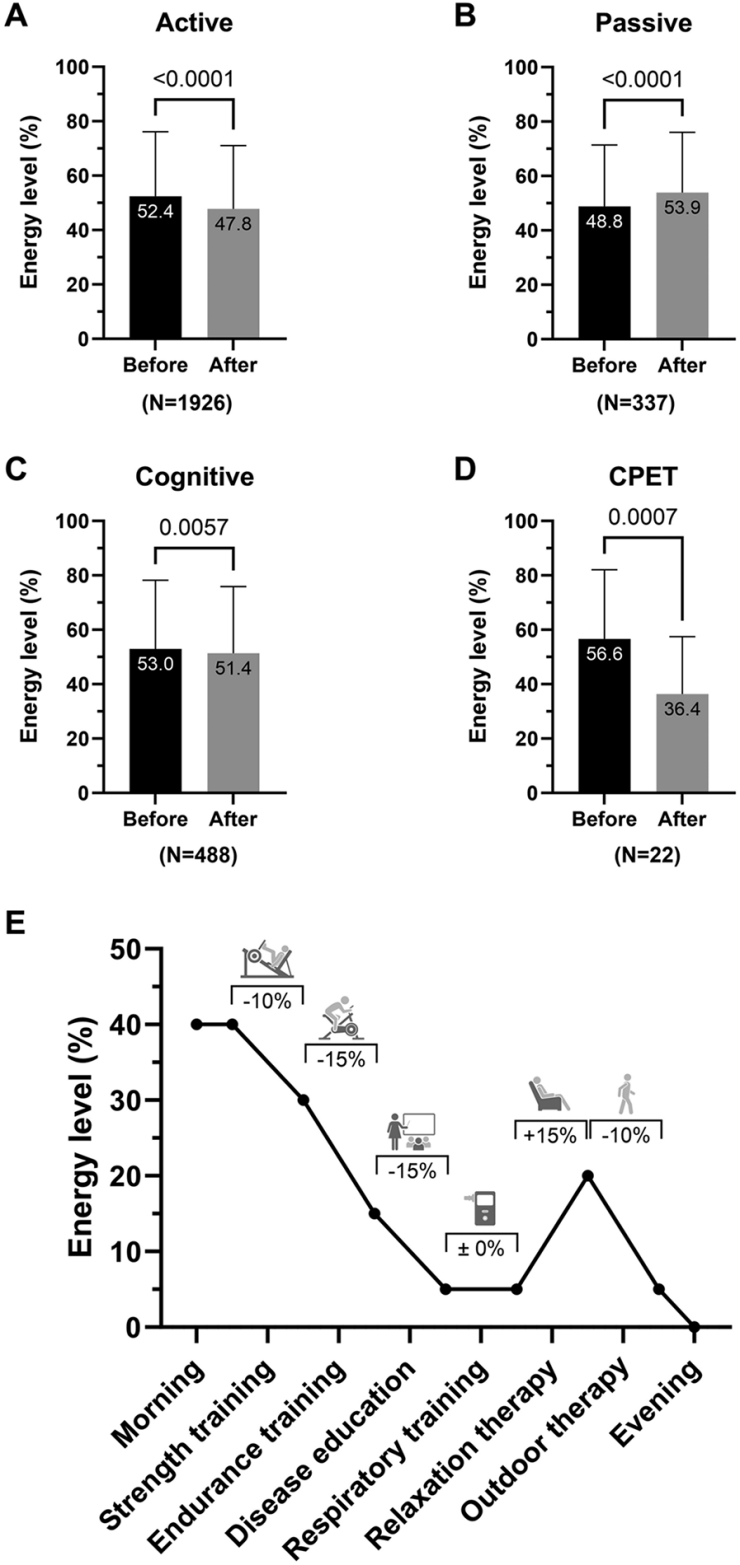
The post-COVID-19 syndrome (PCS) energy diary indicates changes in patients’ energy level subsequent to therapy. **(A–D)** Energy levels before and after individual patient's therapies (*N* = 33) were compared by type of therapy. **(E)** A representative daily progression is depicted with therapies and respective changes in energy level. On average, active therapies reduced energy levels by ∼5% while passive therapies regenerated energy levels to a similar extent. A larger energy drain (∼20%) was seen after cardiopulmonary exercise testing (CPET). In total, PCS patients performed 1,926 active, 337 passive, and 488 cognitive therapies during a mean of 29.9 ± 5.7 rehabilitation days. Active therapies included all physical and respiratory therapies, passive therapies included relaxation therapies, and cognitive therapies included cognitive training, disease education, and talks. CPET was listed separately to analyze the effect of strenuous physical activity on energy levels. Data are presented as mean ± SD and were analyzed using paired *t*-test.

General, mental, and physical fatigue was assessed with the validated MFI-20 questionnaire at admission and discharge and correlated to the mean daily energy levels reported in the diary (Figure 3). On admission, the mean daily energy level of patients with PCS showed an inverse correlation with general (*r* = −0.3591, *p* = 0.0077) and physical (*r* = −0.3229, *p* = 0.0173) but not overall (*p* = 0.067) and mental fatigue (*p* = 0.836). At the end of rehabilitation, all subgroups of fatigue correlated significantly with the energy levels assessed via the diary (all *r* ≤ −0.4018, *p* ≤ 0.0021; Figures 3E–H). The effects of individual therapy classes (active, passive, cognitive) on energy levels were analyzed in patients from center Koenigsfeld (Figure 4; *N* = 33) to assess the clinical applicability of the diary. During a mean of 29.9 ± 5.7 days, patients participated in ∼23 therapies per week including ∼11 active and ∼3 passive therapies. Energy levels decreased on average by ∼2% with each cognitive therapy session (total of 488 sessions, *p* = 0.0057) and by ∼5% with active therapy sessions (1,926 sessions), while passive therapy sessions (337 sessions) regenerated energy levels by ∼5% (both *p* < 0.0001). Of note, cardiopulmonary exercise testing (CPET) was perceived as a strong energy drain, reducing the energy level by ∼20% (*p* = 0.0007). The change in energy levels in percent correlated significantly with the rating of therapies using emojis (*r* = 0.4124, *p* < 0.0001).

## Discussion

The present study aimed to develop a diary to keep record of patients with PCS' energy levels throughout the rehabilitation process and investigate its usability and validity. Our findings suggest that the resulting diary, developed together with affected patients, was able to detect changes in energy levels throughout the day and that reported energy levels correlated significantly with MFI-20 scores of overall, physical and mental fatigue. The diary was able to reflect the effects of the different therapy classes (active, passive, cognitive) on energy levels. The initial diary version was based on the assessment of fatigue levels. Interestingly, this first version was incapable of detecting changes in fatigue perception during the day. The terms “fatigue” and “low energy” are often used equivalently in the literature but must be distinguished in terms of objective and quantifiable changes in performance called fatigability (44, 45). Since no common definition in the scientific domain has been agreed upon (46), it is necessary to subdivide the term fatigue into pathological and non-pathological, physical vs. mental, neurological vs. non-neurological, and cognitive vs. affective fatigue and overlaps (45). At least in neurological diseases such as multiple sclerosis and Parkinson's disease, perception of fatigue and performance fatigability have been shown to be distinct and potentially independent (47, 48). Our data suggests that within this multidimensional condition of constant fatigue as seen in PCS, subjective changes during rehabilitation may be better described as energy gains and losses as indicated by the patients involved in the diary development. The significantly lower energy level in PCS patients compared with CAD patients (∼40% in morning and ∼20% in evening energy level) further emphasized the need for adjustments of rehabilitation programs according to individual energy levels by personalized training loads and strategies, e.g., pacing. The reported mean daily energy levels correlated well with the results of the Multidimensional Fatigue Inventory indicating an overall association of the constructs. The markedly stronger correlations at discharge compared with admission might indicate an improved understanding of pacing with increased awareness of energy resources. Moreover, it might reflect the time-dependent development of such a skill as for other aspects of mindfulness.

The diary was able to detect energy drains and gains: active therapies such as aerobic or strength exercise reduced energy levels while passive therapies such as relaxation therapy regenerated energy levels. The largest energy drain (∼20%) was seen after exhaustive, cardiopulmonary exercise testing (CPET), and might be sex-specific (49). Using the fatigue diary, it was also possible to follow the overnight restoration of energy levels after active therapies and CPET. The observed difference between types of rehabilitation indicates that energy restoration overnight (i.e., after completing daily therapy) may be more successful in inpatient rehabilitation than in outpatient rehabilitation. While this might be explained by travel burden and regular tasks of daily life, further investigations are warranted.

In current clinical management and monitoring of fatigue of different origin, experts mainly use either self-reported fatigue levels such as the visual analog scale, multidimensional fatigue scales such as the MFI-20 or the Functional Assessment of Chronic Illness Therapy Scale—Fatigue, as well as the European Organization for Research and Treatment of Cancer quality of life questionnaire fatigue subscale (EORTC QLQ-C30), among others (50). However, these assessments are episodic, and questionnaires and scales are not designed to assess patients’ symptom changes in everyday life. Moreover, they do not enable patients and therapists to visualize intraday changes or adjust behavior and therapy frequently and in time. We postulate that a PCS-specific diary could enable rehabilitation teams to adjust patients’ therapies quickly and more effectively to individual needs. We believe that energy-tracking is essential for daily performance management and that our data and the developed diary can help to individualize and optimize PCS rehabilitation. With respect to post-rehabilitation and ambulant use, symptom observation by activity logs, diaries, calendars, and smart devices has been suggested to identify potential risk factors for energy loss (51). A digital version of the PCS diary could help to collect long-term data on the possible course of PCS, to identify risk factors for poor progress, and to assess the need for subsequent interventions (52).

A fine tuning of exercise load and exercise capacity seems to be crucial for a positive outcome of exercise therapy in many chronic diseases, either during pacing for patients with PCS/ME/CFS or other chronic neurological/immunological diseases such as multiple sclerosis, Parkinson's disease, rheumatic disease, and cancer (15). Therefore, a broader need for personalized monitoring of energy levels is necessary. Data from CAD patients suggest some general validity of the energy diary and its practical applicability for other diseases with limitations in exercise performance. As expected, morning and evening energy levels were significantly higher compared with PCS patients. The slightly increasing morning energy levels during rehabilitation might indicate a positive adaptation of fatiguability not seen in PCS patients, while changes in exercise capacity during rehabilitation were comparable for CAD and PCS patients (11).

For a successful clinical application, patients' acceptance is of central importance. In this respect, the interview results suggested that keeping a self-management diary was convenient for patients during rehabilitation and was well accepted. Patients also indicated that reporting their feedback to therapists in detail was of importance to them. These results are in line with reports suggesting that a codesign approach including patients' perspectives is useful in the development of tools and technology assisting patients during rehabilitation (53).

The current study has some limitations. While patients kept detailed track of changes in energy levels during therapy, changes during evenings, weekends, and after discharge were not recorded. Thus, no conclusions on the applicability of the diary in daily life can be drawn. Moreover, data from participants after rehabilitation to evaluate the benefits of energy management based on the energy diary would be useful to investigate the long-term effects of the tool.

To conclude, we provide evidence that a diary based on patients' subjective energy assessment represents a useful and valid tool to detect and control the effects of daily therapy sessions during the rehabilitation of patients with PCS. It may be used to facilitate specific scheduling of therapies during rehabilitation and may help implement pacing. The diary may be a useful therapeutic tool for patients living with fatigue, including patients with ME/CFS, cancer, multiple sclerosis, and other immunologic disorders. However, detailed studies on validity in these patient groups are still necessary.

## Data Availability

The raw data supporting the conclusions of this article will be made available by the authors, without undue reservation.
