# Supplementary material for: Design and validation of an energy level diary for fatigue management in patients with post-COVID syndrome
Source: Front Rehabil Sci. 2025 Jul 21;6:1633466. doi: 10.3389/fresc.2025.1633466 (PMC12318978; doi:10.3389/fresc.2025.1633466)
Supplement: Supplementary file 1 [file Table1.docx]

**Supplementary Material**

**Suppl. Figure 1: Final version of energy diary** including spaces to indicate therapy session type, energy levels

(beginning of the day, after each therapy session, at the end of the day), and color-coded emojis.

**
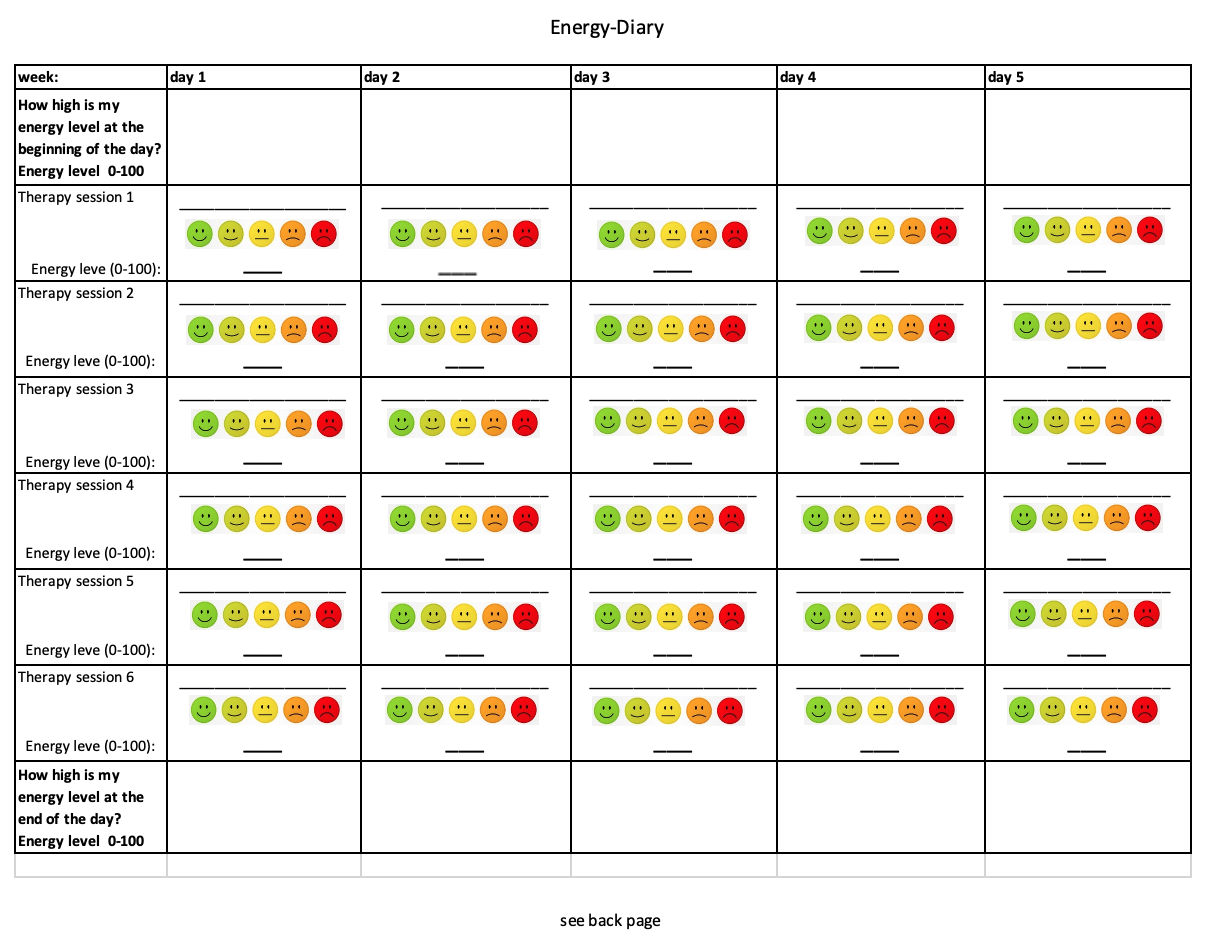
**

**Supl. Table 1: Interview results**

Each interview began with an introduction with the following words: **“***This is an interview by which we want to learn from you how to improve the Energy Diary that we handed out to the you during your rehabilitation. This will help us to improve and make enhancements in the layout, wording, and content of the diary for you and other patients. You are a valuable part of this process, and we appreciate your opinion. We won’t judge you and your participation will not negatively affect your future therapies.”* Afterward, the following interview questions were presented:

| **No.** | **Question** | **Answer categories** | **Examples of answers** |
| --- | --- | --- | --- |
| 1 | Was the design appropriate for a diary (formatting, color features, font size)? | Yes: N=19 (95%)  No: N=1 (5%) | *“The diary has a functional design which helps me to use it as a tool for self-reflection”* |
| 2 | Was there sufficient time to fill in the diary during the rehabilitation day? | Yes: N=17 (85%)  No: N=3 (15%) | *“I had enough time to fill out the diary, but I’d like to have more space for personal notes* |
| 3 | What helped you more in assessing your energy level at the beginning of the day, after each exercise and at the end of the day, the smiley faces (visual tracking) or the classification into energy levels (percentual tracking), both or none of both? | Percentual tracking via energy level 0-100’ helped me more to assess my energy level: N=11 (55%)  Visual tracking with smileys helped me mor to assess my energy level: N=4 (20%)  Both percentual and visual tracking helped me equally to assess my energy level: N=3 (15%).  Neither percentual nor visual tracking helped to assess my energy level: N=2 (10%) | *“Dividing energy levels into 0-100 was a little challenging at the beginning, but became easy later on and felt very accurate to express one’s energy level.”*  *“The colors of the smileys helped me to quickly evaluate my physical and mental state in that moment.”* |
| 4 | Was the diary helpful to learn the process of pacing (e.g. what gives or drains energy? What effects do therapies have?) | Yes: N=19 (95%)  No: N=1 (5%) | *“The diary helped me to identify and understand how cognitive or physical effort lead to fatigue.”* |
| 5 | Would you accept the diary in its existing format or are there major changes needed? | Yes, I accept the diary in its existing format: N=19 (95%).  No, major changes are necessary: N=1 (5%) | *“There is not enough space for personal notes and comments.”* |
| 6 | Would you like optional additions?  (examples: space for entries at home/weekends, more space for individual entries and symptoms) | Yes, I would like optional additions: N=13 (65%)  No, there are no additions necessary: N=7 (35%) | *“I missed space for entries after the rehabilitation day.”* |
| 7 | Did you use the diary after rehabilitation at home? | Yes, I used the diary at home: N=7 (35%)  No, I did not use the diary at home: N=13 (65%) | *“I used the diary in everyday life for a short while, but then I was not disciplined enough to continue.”* |
| 8 | Would you prefer a digital form? | Yes: N=11 (55%)  No: N=9 (45%) | *“I would prefer a digital solution, since it would be available anytime, anywhere.”* |
| 9 | Do you have any more ideas or further suggestions? | Yes: N=0 (0%)  No: N=20 (100%) |  |
